# The Effect of Digital Era on Human Visual Working Memory

**DOI:** 10.1002/brb3.70220

**Published:** 2024-12-31

**Authors:** Dandan Tang, Jiangtao Chen, Ping Xu

**Affiliations:** ^1^ School of Psychology Fujian Normal University Fuzhou China; ^2^ College of Educational Science Zhoukou Normal University Zhoukou China; ^3^ Research Center of Brain and Cognitive Neuroscience Liaoning Normal University Dalian China; ^4^ Educational Psychology Division, Public Course Teaching Department Guangzhou Sport University Guangzhou China

**Keywords:** active video games, digit age, digital content, virtual reality, visual working memory

## Abstract

**Background:**

The digital age has had a profound impact on our lives and cognitive abilities, such as working memory. Typically, visual working memory (VWM) is an important aspect of our working memory. As a crucial cognitive function for individuals, VWM has been extensively studied in the context of the digital age and may be affected by the digital age.

**Objective:**

This review aims to provide a summary of the impact of the digital age on VWM and cover various aspects and novel methods for investigating its effects on our VWM.

**Methods:**

Qualitative review of the VWM in the context of the digital age.

**Results:**

This paper reviews the research on VWM in the context of the digital era, expounds the influence of both the digital content usage and the active video games on the VWM, introduces the application of the virtual technology in the research of VWM, and puts forward the future research direction of VWM in the context of the digital era.

**Conclusion:**

By synthesizing the existing research, this review sheds light on the complex relationship between the digital age and VWM, as well as identifies potential avenues for future research on VWM.

## Graphical abstract

Our visual working memory (VWM) has been affected by the digital age. The present paper qualitatively reviews the VWM research works in the context of the digital age and reveals the complex relationship between the digital age and VWM, as well as puts forward the future research direction of VWM.

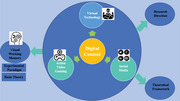

## 1 Introduction

The contemporary era is characterized by the widespread adoption of digital technology, which has had a profound influence on human cognition. Of particular interest is the examination of how digital technology affects visual working memory (VWM). VWM is a fundamental cognitive process involved in the temporary storage and manipulation of visual information, supporting various tasks such as object recognition, spatial navigation, and mental imagery (Baddeley 1992). The ubiquitous use of digital technology has resulted in individuals encountering increasingly fragmented and complex visual stimuli. Moreover, there have been notable shifts in how visual information is encoded and retained, such as the transition from reading printed materials to consuming digital texts on devices like smartphones and computers. These factors are significant contributors to the impact of digital technology on VWM. While some research suggests that digital technology can enhance VWM by providing immersive and interactive experiences that demand attention and memory skills, it can also have deleterious effects by overloading VWM with excessive and irrelevant information.

This narrative review aims to provide a comprehensive overview of the influence of digital technology on VWM, with a specific focus on media usage, active video games, and novel methodologies employed to investigate their effects. We have searched the relevant subject about digit content and VWM using the databases such as PubMed, ProQuest, Elsevier, ERIC, and Web of Science with the keywords “visual working memory,” “digit content,” “active video games,” “virtual reality,” and “digit age.” The last literature search was conducted in February 2024. By synthesizing the existing literature, this review seeks to shed light on the intricate relationship between digital technology and VWM while identifying potential avenues for future research.

## 2 Influence of Digital Content Usage on VWM

Social media has become an integral part of modern society, providing constant access to information, entertainment, and interpersonal connections. It is nearly ubiquitous, with individuals rarely being separated from their devices.

Our review of the literature on digital media's impact on VWM reveals a variety of research methodologies. One such method is the *n*‐back task, a standard measure of WM capacity where participants indicate whether a given stimulus matches one presented ‘N’ trials earlier. For example, in a 2‐back task, participants must identify if the current stimulus is the same as the one shown two trials before. Numerous studies have investigated the impact of social media usage on cognitive performance, particularly in relation to VWM. However, findings in this area have been inconsistent. One study suggests that the mere presence of social media usage or smartphone usage can diminish available cognitive capacity, with the highest cognitive costs observed among individuals who exhibit greater dependence on smartphones (Ward et al. 2017). Additionally, previous research indicates that separation from one's smartphone can have negative effects on mental shifting, inhibitory control, and working memory (WM), which are mediated by feelings of anxiety (Hartanto and Yang 2016). Notably, individuals displaying high symptom severity of what researchers refer to as “smartphone addiction” performed poorly in inhibitory control tasks. Moreover, social media usage has been linked to adverse emotional well‐being and psychological disorders (Woods and Scott 2016; Austermann, Thomasius, and Paschke 2021), which may contribute to the negative effects on cognitive performance or WM (Sharifian and Zahodne 2021). However, contrasting findings exist, as some studies have reported no differences in WM between habitual and non‐habitual social media users (Hainš, Kućar, and Kovačić 2020). In fact, certain research even suggests that social media usage may improve WM performance in healthy older adults (Myhre, Mehl, and Glisky 2017).

Moreover, Almarzouki et al. (2022) conducted a study investigating the mediating role of WM in this relationship between social media usage and psychological factors such as depression, anxiety, and disordered social media use. The findings indicated no significant difference in WM performance between participants in the social media condition and in the control conditions. However, among individuals with moderate levels of depression, social media usage was associated with significantly more errors. Additionally, higher scores on depression measures and greater habitual social media usage were predictive of higher levels of disordered social media use, while academic performance did not exhibit a significant relationship with WM or social media usage. In a different study, Sternberg, Luria, and Sheppes (2018) explored the moderating role of neural WM in the filtering of Facebook information and its association with symptoms of anxiety. The results demonstrated that increased usage of Facebook was linked to greater anxious symptoms in individuals with impaired Facebook filtering ability, suggesting that neural social network filtering might serve as a potential moderator in comprehending the adverse effects of heightened online social network usage on mental health. Another study investigated the impact of distractions caused by mobile instant messaging, specifically the WhatsApp application, on WM performance among teenagers. The findings revealed that engaging in WhatsApp distractions resulted in decreased WM performance, and the students themselves were cognizant of the detrimental effects of WhatsApp usage on learning effectiveness (Aharony and Zion 2019).

In addition to the social media usage, there were studies of interest in the relationship between media multitasking and WM. Media multitasking involves engaging in multiple forms of media simultaneously, such as listening to music while writing. Previous research has primarily adopted an individual‐differences approach to investigate people's tendency to engage in media multitasking in their daily lives (Ophir, Nass, and Wagner 2009). It has been found that heavy media multitaskers tend to perform poorly in tasks involving WM (Minear et al. 2013). Subsequent studies found that heavy media multitaskers exhibited lower WM performance regardless of the presence or absence of external distractions. Differences in memory among media multitaskers appear to be related to discriminability rather than decision bias. Furthermore, attentional impulsivity has been found to correlate with media multitasking behavior and reduced WM performance (Uncapher, Thieu, and Wagner 2016). Another study, using a correlational approach and treating media multitasking as a continuous variable, investigated its relationship with a specific WM paradigm, namely the *n*‐back task. The findings revealed that higher scores on media multitasking were associated with a greater number of omitted trials in the 2‐back and 3‐back tasks, suggesting that heavy media multitaskers may be more disengaged during these tasks (Ralph and Smilek 2017). A recent study, after controlling for age, IQ, and attentional impulsivity, found a marginally significant association between higher levels of media multitasking and greater WM capacity scores (Murphy and Creux 2021). Overall, these findings suggest that chronic media multitasking is linked to a broader attentional scope and higher attentional impulsivity, which may result in increased competition between goal‐relevant and goal‐irrelevant information.

In addition, in recent years, a significant shift toward remote work, virtual education, and social distancing measures was witnessed, thereby leading people to increasingly depend on digital devices for communication, work, and entertainment. This increased exposure to screens has raised concerns regarding its potential negative impact on cognitive function. A study revealed that participants who had higher night screen exposure had lower cognitive scores in the information speed processing, WM, calculation, and attention domains (Shalash et al. 2024).

This divergence in research outcomes indicates that the influence of digital media on VWM is not uniform. For instance, the cognitive load experienced during multitasking and media use, as highlighted by Ward et al. (2017), may detract from VWM performance. In contrast, the cognitive exercise provided by specific types of games, as demonstrated by Jakubowska et al. (2021), could potentially hone cognitive functions. Furthermore, studies that report no significant impact of gaming on VWM capacity suggest that other factors, such as individual differences, genre of games, and usage habits, may play a pivotal role in this dynamic.

To achieve a comprehensive understanding of how digital media usage affects VWM, future research should delve deeper into how different media types, patterns of usage, and individual characteristics interplay to influence cognitive functions. Moreover, considering the significance of VWM in everyday life, these research outcomes have important practical implications for the design of more effective cognitive training programs and the optimization of digital media usage habits.

## 3 Influence of Active Video Game on VWM

Action video gaming (AVG) has gained popularity as a form of gaming that involves processing multiple complex visual stimuli and responding to them within strict time constraints (Green and Bavelier 2012). This type of gaming relies on various cognitive abilities, including visual processing, attention, WM, multitarget‐tracking skills, and inhibitory control (Green and Bavelier 2007, 2012; Spence and Feng 2010). VWM is a critical cognitive function that allows individuals to acquire knowledge from the visual environment. Its limited capacity, estimated to be approximately three or four items (Alvarez and Cavanaugh 2004; Luck and Vogel 1997), highlights the need to investigate whether it is possible to enhance VWM via training. The color wheel task is another tool used to assess attention and memory, requiring participants to track a color on a rotating wheel, which tests their ability to retain and process visual information.

Previous research has demonstrated that individuals who engage in traditional AVG (AVGPs) outperform nonvideo game players (NVGPs) in tasks assessing visual short‐term memory (VSTM), such as change detection, color wheel tasks, scene change detection, and enumeration tasks (Boot et al. 2008; Sungur and Boduroglu 2012; Clark, Fleck, and Mitroff 2011; Green and Bavelier 2006; Blacker and Curby 2013; Wilms, Petersen, and Vangkilde 2013; Blacker et al. 2014; Li et al. 2015; Colzato et al. 2013; Oei and Patterson 2013; McDermott, Bavelier, and Green 2014; Waris et al. 2019). Furthermore, recent investigations have explored the potential cognitive benefits of action real‐time strategy gaming (ARSG), a relatively new genre of AVG that combines action and strategy elements, necessitating timely strategic decision‐making and teamwork. Due to its high cognitive demands, ARSG provides a promising avenue for studying cognitive and neural plasticity. ARSG shares similarities with AVG in terms of sensorimotor skills and action‐oriented gameplay (Dale and Green 2017a, 2017b; Bavelier and Green 2019; Dale et al. 2019). Recent studies employing behavioral and event‐related potential measures have indicated that ARSG experts exhibit higher accuracy and larger VWM capacity compared to non‐experts (Qiu et al. 2018; Gan et al. 2020; Yao et al. 2020). Moreover, these findings suggest that prolonged ARSG experience may enhance VWM, offering new insights into cognitive and neural plasticity (Gong et al. 2015, 2016, 2017; Kowalczyk et al. 2018; Gong, Li, et al. 2019; Gong, Yao, et al. 2019; Yao et al. 2020). Additionally, studies have demonstrated that ARSG gameplay is associated with increased theta‐band power in EEG, which is related to WM load, as well as improved visual attention—both factors linked to individual differences in VWM capacity (i.e., life simulation gaming; Gong, Li, et al. 2019).

Furthermore, recent investigations have examined the effects of playing real‐time strategy (RTS) video games on cognitive development, specifically on VWM capacity. In one study, participants without prior video game experience were randomly assigned to an experimental group that received 30 h of training in a variable environment, an active control group that received training in a fixed environment, or a passive control group. Both experimental groups engaged in playing StarCraft II, an RTS video game. The findings revealed significant increases in VWM capacity among the experimental groups after training, compared to the control group. Moreover, the study observed a relationship between the psychophysiological marker of VWM capacity and the extent of improvement in the RTS video game, but solely within the experimental group. These results suggest that the psychophysiological marker of VWM capacity may serve as a predictor of future proficiency in video game training (Jakubowska et al. 2021). In another study, researchers explored the influence of action video game playing on susceptibility to distraction and investigated whether this effect was attributable to enhanced VWM capacity. The results indicated that action video game players (AVGPs) did not exhibit reduced susceptibility to distraction compared to non‐action video game players. However, individuals with higher VWM capacity demonstrated lower susceptibility to distraction, irrespective of their video game experience. These findings imply that VWM capacity may have a stronger association with resistance to distraction than video game expertise (Hauck and Lien 2022).

Action video gaming (AVG) requires the rapid processing of complex visual stimuli, which might lead to a more robust visual memory representation that is less susceptible to external interference, and the AVG experience has been shown to enhance VWM capacity (Green and Bavelier 2012). The cognitive demands of AVG, such as the need for visual processing and inhibitory control, may stimulate neuroplastic changes that improve VWM (Dale and Green 2017a). However, the relationship between video gaming and VWM is not straightforward, as other studies have found no significant difference in VSTM capacity between gamers and non‐gamers (Wilms, Petersen, and Vangkilde 2013). This suggests that while the AVG may enhance certain perceptual and cognitive functions, it may not directly increase the capacity of VSTM, and the impact of AVG on VWM may be moderated by some factors such as the type of game, the duration of play, and the individual's baseline cognitive abilities. In summary, the link between video gaming and VSTM is complex, which could be due to variations in study design, participant selection, task types, and the diversity of gaming experience.

Collectively, these findings suggest that engaging in AVG playing may enhance cognitive development, particularly in the realm of VWM capacity holding promise for the potential utilization of AVG as tools for cognitive training and rehabilitation. Nevertheless, further investigation is required to elucidate the underlying mechanisms of this relationship, as a separate study found no significant difference in visual short‐term memory (VSTM) capacity between individuals who play action video games and those who do not (Wilms, Petersen, and Vangkilde 2013). Consequently, the question regarding whether AVG players possess an advantage in VSTM capacity remains unresolved and necessitates additional examination.

## 4 Using Virtual Technology to Investigate VWM

The integration of virtual reality (VR) technology into the study of visuospatial working memory (VWM) offers a unique opportunity to explore cognitive processes in a more naturalistic and interactive environment akin to digital media usage. VR allows researchers to investigate the theoretical aspects of VWM, such as the maintenance and selection of visual information during navigation and self‐movement, which are increasingly relevant in the digital age. Studies like those by Blacker et al. (2017) and Draschkow, Kallmayer, and Nobre (2021) have utilized VR to reveal the importance of VWM in spatial navigation and the integration of spatial relations, skills that are critical for navigating today's digital landscapes.

Moreover, VR has been effectively applied in interventions aimed at enhancing VWM and attention, as demonstrated by Coleman et al. (2019). This article discusses the effectiveness of computerized cognitive interventions, specifically WM training, on improving ADHD‐related inattention and off‐task behavior in children. The study involved 15 children aged 6–15 with attention problems who underwent 5 weeks of Cogmed WM training and were assessed using the Virtual Classroom Continuous Performance Task (VCCPT), a VR‐based measure of sustained and selective attention, before and after the training. The results indicated improvements in several areas of attention performance, such as reduction in omission errors, faster reaction times, and decreased reaction time variability. The study suggests that WM training can lead to significant enhancements in sustained attention within real‐life classroom settings. Furthermore, the use of the VCCPT as a psychometrically validated VR measurement tool provides additional validity compared to teacher or parent reports. These findings contribute to the debate on evaluating the effectiveness of WM training in relation to real‐life improvements and help inform consumer understanding of such interventions.

Their use of a VCCPT to measure the effects of WM training on attention performance highlights VR's potential in developing ecologically valid assessments and interventions (Ballard, Hayhoe, and Pelz 1995; Hollingworth 2004).

In conclusion, the use of VR in studying VWM not only provides a more realistic experimental paradigm that mirrors digital media environments but also opens up new avenues for developing interventions to improve cognitive functions affected by digital media usage. By connecting VR research to the broader theme of digital media's impact on VWM, we can gain a deeper understanding of the cognitive processes involved and the potential for targeted cognitive training programs.

## 5 Theoretical Framework

The influence of digital content on VWM is grounded in cognitive theories that emphasize the role of attention and cognitive control in memory processing. The cognitive load theory posits that digital media, with its multifaceted and often complex information, can overwhelm the limited capacity of WM (Sweller 1988). Our review integrates this theory to explain the observed effects of digital media on VWM. Additionally, we consider the impact of emotional well‐being and mental health on cognitive performance, drawing on research that links stress and anxiety to impaired cognitive control and attention. These factors are crucial for the effective functioning of VWM, and their interplay with digital media usage is a key area of exploration in our review.

In addition, studies suggest that the mere presence of social media or smartphone usage can diminish cognitive capacity, particularly among individuals with high dependence on smartphones (Ward et al. 2017). This aligns with the cognitive load theory, as the constant influx of information from social media may tax the limited resources of VWM. Conversely, some studies report no difference in WM performance between habitual and non‐habitual social media users (Hainš, Kućar, and Kovačić 2020), or even improvements in certain populations, such as healthy older adults (Myhre, Mehl, and Glisky 2017). These discrepancies may be attributed to individual differences in cognitive abilities and emotional resilience, as well as the specific content and context of social media usage. Moreover, media multitasking, the simultaneous engagement with multiple media forms, has been linked to poorer performance on tasks involving VWM (Minear et al. 2013). This relationship is further complicated by the finding that heavy media multitaskers exhibit lower WM performance regardless of external distractions (Uncapher, Thieu, and Wagner 2016). We propose that media multitasking may tax the attentional control necessary for effective VWM, leading to reduced cognitive performance. However, the relationship is not uniform, as some studies suggest a positive association between media multitasking and WM capacity (Murphy and Creux 2021). This may indicate that certain individuals possess the cognitive flexibility to effectively juggle multiple information streams, enhancing their VWM capacity. Therefore, the impact of digital media on VWM is multifaceted, involving multiple factors such as cognitive load, emotional health, individual differences, and media multitasking behavior. Future research is needed to explore the interactions between these factors and their specific effects on VWM in more depth.

## 6 Potential for Future Research in VWM in the Digital Era

In today's digitally oriented society, VWM assumes a critical role in the maintenance and manipulation of visual information within brief time periods. Given the increasing pervasiveness of digital technology, it is imperative to investigate the potential impact of digital media on VWM. This entails examining how different forms of digital media, including text, images, and video, influence VWM capacity and precision. Furthermore, understanding the developmental implications of digital media on VWM in children and adolescents at various stages of cognitive maturation is crucial.

Several research inquiries emerge as significant in this domain. Firstly, investigating the effectiveness of training programs or interventions designed to enhance VWM within the context of digital media usage is warranted. Additionally, comprehending how digital media utilization interacts with other cognitive processes such as attention, decision‐making, and problem‐solving represents an essential avenue for exploration. Moreover, it is imperative to examine whether individual differences, such as age, cognitive ability, or prior experience with digital technology, moderate the effects of digital media on VWM.

## 7 Conclusions

The present digital age has had a significant impact on our cognition, especially on our VWM. By revealing the relationships between the digital age and human VWM, and the effect of the digital age on human VWM, we propose that a more comprehensive understanding of the impact of digital media on VWM can be attained. Consequently, this review contributes to the development of strategies aimed at optimizing VWM functioning within the digital era.

## Author Contributions

**Dandan Tang**: conceptualization, supervision, writing–review and editing, methodology, validation, formal analysis, project administration, writing–original draft. **Jiangtao Chen**: methodology, formal analysis, writing–original draft, software, data curation, writing–review and editing. **Ping Xu**: supervision, writing–review and editing, funding acquisition, validation, conceptualization, methodology, project administration, resources.

## Conflicts of Interest

The authors declare no conflicts of interest.

### Peer Review

The peer review history for this article is available at https://publons.com/publon/10.1002/brb3.70220.

## Data Availability

Data sharing not applicable to this article as no datasets were generated or analyzed during the current study.
